# Research on Tail Rotor Load Test Flight Technology for Helicopters Based on Strain Sensor Measurement

**DOI:** 10.3390/s26082287

**Published:** 2026-04-08

**Authors:** Shuaike Jiao, Jiahong Zheng, Kang Li, Xiaoqing Hu

**Affiliations:** 1 Chinese Flight Test Establishment, Xi’an 710089, China; 040715316@163.com (J.Z.); lk809450704@163.com (K.L.); huxq15619203852@163.com (X.H.); 2 Shananxi Aircraft Structural Integrity Flight Test Technology Innovation Center, Xi’an 710089, China

**Keywords:** the tail rotor load, flight test, Wheatstone bridge, helicopter load risk test point matrix

## Abstract

The load characteristics of the helicopter tail rotor system are critical to flight safety and handling performance, and flight testing remains the most direct and reliable means to obtain authentic load data. In this paper, the well-established Wheatstone bridge strain measurement method is adopted to carry out accurate load testing on the helicopter tail rotor system. The tail rotor assembly mainly consists of the tail rotor shaft, pitch link, and tail rotor blades, which undertake different load transfer tasks during flight. Under actual operating conditions, the tail rotor shaft bears significant axial tension as well as combined lateral and vertical bending moments; the pitch link is primarily subjected to alternating axial tension and compression; and the tail rotor blades withstand complex loads including flapping bending, lagwise bending, and torsional moments. According to the distinct stress characteristics and force transmission paths of each component, targeted flight test maneuvers are reasonably designed. These maneuvers include steady-level flight at low, medium, and high speeds, zigzag climbing flight, near-ground side-rear flight, as well as deceleration-to-sprint and obstacle slope maneuvers specified in ADS-33E. Key flight parameters are selected for in-depth analysis to reveal the load distribution and dynamic variation patterns of the tail rotor under typical operating conditions. On this basis, a helicopter load risk test point matrix is established to identify high-risk working conditions and key monitoring positions. This study provides a solid theoretical and data foundation for subsequent flight test monitoring and structural strength verification. It effectively reduces flight test risks, improves monitoring efficiency and accuracy, and helps cut down the human, material, and financial costs associated with flight test monitoring. The research results can also provide important references for the design optimization and safety evaluation of helicopter tail rotor systems.

## 1. Introduction

The tail rotor is a critical aerodynamic component of helicopters, serving two primary functions: counteracting the main rotor’s torque in single main rotor helicopters, and providing yaw stability to enable directional control. The load characteristics of the tail rotor directly impact flight safety and operational performance. As helicopters evolve toward higher speeds and greater payload capacities, the tail rotor system faces increasingly complex dynamic load environments. Traditional design methods and ground tests have become insufficient for comprehensively evaluating its actual in-flight load characteristics. Statistics show that approximately 28% of global helicopter accidents in the past decade were related to tail rotor system failures, with most incidents revealing significant discrepancies between predicted and actual flight loads.

To address this issue, numerous researchers have investigated tail rotor loads. In 2006, J. G. Leishman [1] described helicopter tail rotor aerodynamics in the second edition of Principles of Helicopter Aerodynamics. In 2022, W. Johnson [2] analyzed various load measurement methods in AIAA Journal, while H. kim et al. [3] presented a 256-channel FBG demodulation system for tail rotor load testing on UH-60 helicopters in Measurement Science and Technology. That same year, Zhang Mingyuan et al. [4] also conducted dynamic load measurements using Fiber Bragg Grating (FBG) technology. Bell Helicopter patented an FBG-based tail rotor load measurement system [5]. For accurate load prediction, R. Smith et al. [6] employed machine learning techniques. Relevant technical descriptions also appear in the FAA’s Advisory Circular 29-2D: Transport Category Rotorcraft Certification [7], ASA’s CR-202301256:Wireless Rotor Load Monitoring [8], and Airbus Helicopters’ H160 Tail Rotor Flight Test Report [9]. Zhang H L et al. [10] constructed an airborne dynamic strain measurement system using Fiber Bragg Grating (FBG) sensors, and solved the signal transmission problem between the rotating parts of the rotor and the cabin by using power line communication and orthogonal frequency division multiplexing modulation technology. Verified by flight tests, this system can stably acquire dynamic strain data of helicopter rotor blades in harsh environments with strong electromagnetic interference and high vibration, promoting the leap of rotor strain measurement from electrical sensing to optical sensing. Deng Fuwei [11] took the main rotor of a large helicopter as the research object, carried out flight tests using the resistance strain gauge electrical measurement method, covering typical flight conditions such as hovering and high-speed forward flight, systematically analyzed the time-domain and frequency-domain characteristics of main rotor loads as well as the influence laws of parameters such as flight speed and control input, and provided measured basis for the structural design of large helicopter rotors. Zheng Jiahong et al. [12] addressed the problem of coupled measurement of tensile, bending, and torsional loads on the rotor shaft, realized load decoupling by optimizing the layout and configuration of strain bridges, established a reliable flight test measurement method for rotor shaft loads through ground calibration and flight verification, and clarified the load characteristics under severe working conditions such as level flight and slope landing. In terms of load analysis and prediction, Wang Zefeng et al. [13] proposed a method for inversing rotor aerodynamic loads based on measured structural loads. By constructing an inversion model between structural loads and aerodynamic loads, it solved the problem that traditional flight tests are difficult to directly obtain aerodynamic distributed loads. Wu Huifeng et al. [14] used neural networks to fit the nonlinear mapping relationship between flight parameters and blade loads, constructed a high-precision load prediction model, and provided a data-driven idea for rapid estimation of rotor loads. Zhang Junnan et al. [15] developed a helicopter rotor flight load prediction method based on RBF neural networks, which can realize online or offline load prediction without strain measurement, and is suitable for flight test load monitoring and load database construction. In the research on measurement methods and special configurations, Liu Mingming et al. [16] summarized and compared various rotor blade load measurement methods such as the strain electrical measurement method and the optical fiber sensing method, analyzed the advantages, disadvantages and engineering application scenarios of various methods, and provided reference for the selection of flight test measurement schemes. Cheng Weizhen [17] aimed at the aerodynamic interference characteristics of coaxial dual rotors, obtained the load characteristics of upper and lower rotor blades through flight tests, revealed the influence laws of dual rotor interference on loads, and supported the design of coaxial helicopter rotors. Zhang Honglin et al. [18] further optimized the distributed FBG sensing technology, realized the measurement and engineering modeling of rotor dynamic loads combined with the blade dynamic model, and formed an integrated optical measurement and modeling scheme through flight verification, providing reliable support for the engineering application of rotor dynamic loads. The above studies have improved the helicopter rotor load research system from multiple dimensions such as measurement technology, modeling methods, and working condition characteristics, and provided important theoretical and engineering basis for rotor structural optimization, health monitoring, and reliability improvement. This study [19] takes the supercritical helicopter tail transmission system as the research object, and focuses on the dynamic characteristics of the system under the coupling effect of self-excited vibration and rubbing impact. The research aims to reveal the coupling vibration mechanism, identify the key influencing factors, and provide theoretical support for the vibration and noise reduction and structural optimization of the system.

Most of the aforementioned studies were based on laboratory ground bench tests or employed FBG technology that remains relatively immature for engineering applications, with limited research utilizing actual flight test data. This study leverages real flight test data and employs the well-established Wheatstone bridge measurement method [20,21] for tail rotor load measurement. The load distribution patterns and those variation characteristics with relevant parameters are analyzed during typical helicopter maneuvers in this study. Furthermore, a helicopter load risk test point matrix was developed, which can provide data support for subsequent flight test monitoring. This approach reduces flight test risk, enhances monitoring efficiency, and decreases expenditure of human, material, and financial resources.

## 2. Force Analysis of Components in the Tail Rotor System

**Figure 1:**
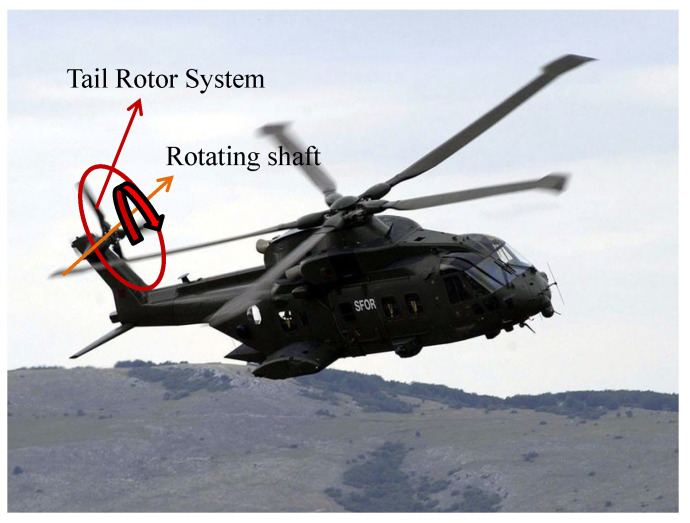
The tail rotor system.

This study focuses on the tail rotor system of the conventionally configured helicopter (single-rotor helicopter with tail rotor). The tail rotor system primarily comprises the tail rotor blades, flexible beams, tail rotor hub, yoke, and tail rotor pitch link. The layout of the tail rotor system on the helicopter is shown in Figure 1.

**Figure 2:**
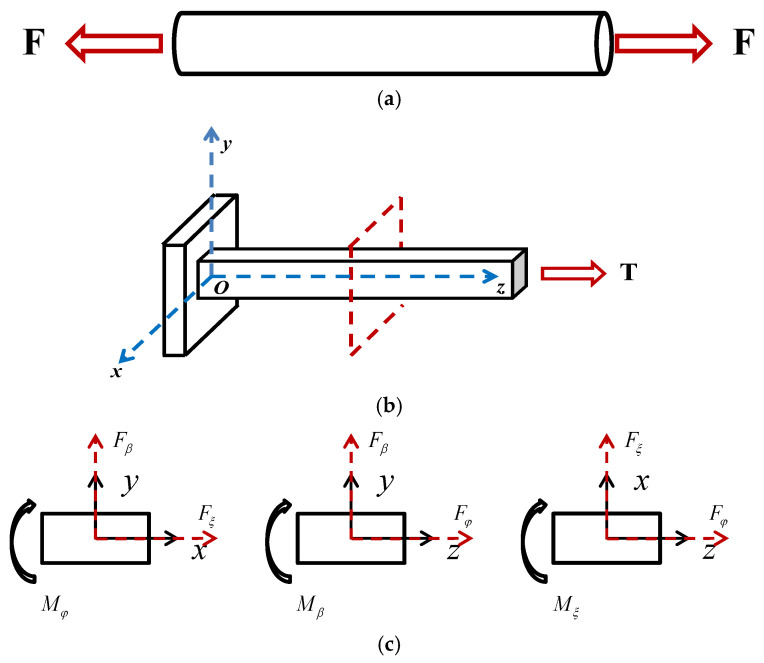
(**a**) The tail rotor pitch link equivalent model diagram. (**b**) The rotor blades or flexbeam equivalent model diagram. (**c**) The free body diagram (FBD) of the rotor blades or flexbeam.

During flight, the primary components of the tail rotor system, the tail rotor blades, flexible beams, and tail rotor pitch links bear the loads. The tail rotor pitch links can be considered equivalent to two-force members, and the flexible beams or tail rotor blades can be equivalently modeled as cantilever beams. The force analysis diagram of the tail rotor system is shown in Figure 2. The equivalent model diagram of the tail rotor pitch link is shown in Figure 2a. As can be seen from the figure, the tail rotor pitch link is only subjected to an axial load F. Mechanical analysis of a given cross-section in the tail rotor blade or flex beam assembly indicates that it is subjected to three forces and three moments, shear force $F_{\beta }$, shear force $F_{\xi }$, fictitious force $F_{\varphi }$, flapwise bending moment $M_{\beta }$, edgewise bending moment $M_{\xi }$ and torque $M_{\varphi }$, which is shown in Figure 2b,c. In normal helicopter operation, because the centrifugal force acting on the rotor blades and flexbeam remains essentially constant and its static strength design margin is sufficient, this force does not contribute fatigue damage. Therefore, centrifugal force is not measured in this study.

In summary, the forces that must be measured for the tail rotor system are the axial force of the tail rotor pitch link and the forces/moments (flapwise bending moment, lead-lag bending moment and torque) on the blades or flexbeam.

## 3. Testing Methods and Equipment

### 3.1. Testing Methods

The load of the tail rotor system is measured by the established electrical resistance strain gauge measurement method in this study. The flapwise of the tail rotor blades or flexbeams are measured using parallel-grid strain gauge; the axial loads on the tail rotor pitch link are measured using orthogonal strain gauge rosettes; lagwise bending moments of the tail rotor blades are measured using single-element strain gauge. The strain gauges, whose model is BA350-2AA(9)-G150-JQC, used in this study were manufactured by AVIC ZEMIC (Hanzhong, China). The strain gauges substrate is metal foil, so its stiffness is better. It can operate on tail rotor blades prolonged service life. The fundamental parameters of the strain gauges are shown in Table 1.

The Wheatstone bridge adopts a DC excitation mode with an excitation voltage of 5 V. The voltage signal of the bridge is transmitted to the collector in the engine room through a small telemetry method. The KM500 collector is a general-purpose airborne collector purchased from Europe, which uses a differential amplifier. We sincerely apologize that our experiment is only used for data collection and recording, and the specific internal circuit has not yet been studied. In addition, the KM500 collector also performs smooth filtering processing on the collected data.

**Figure 3:**
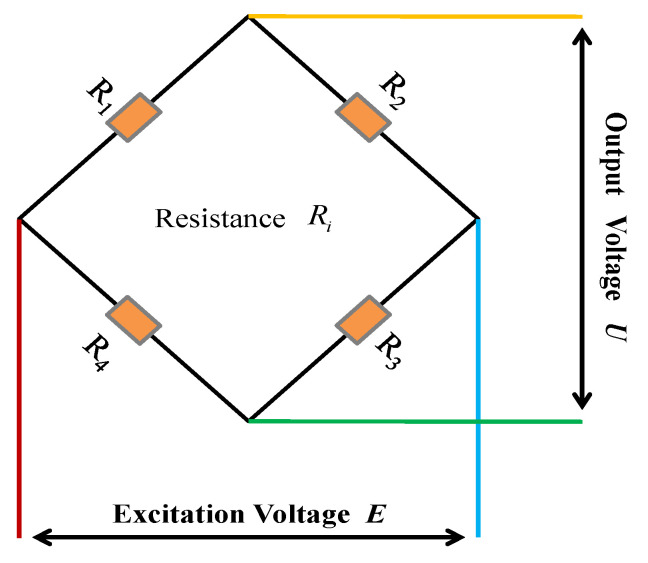
Wheatstone bridge.

The selected strain gauges were connected into a bridge circuit according to Figure 3.

**Figure 4:**
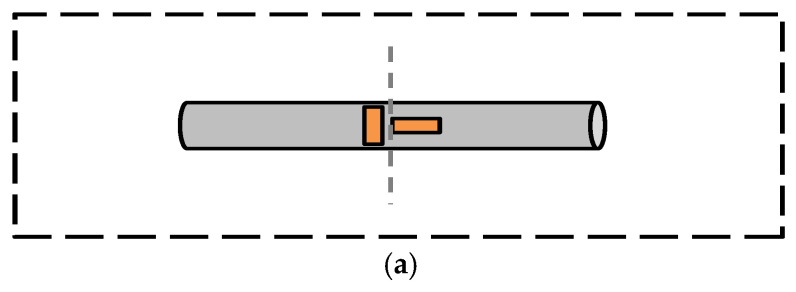

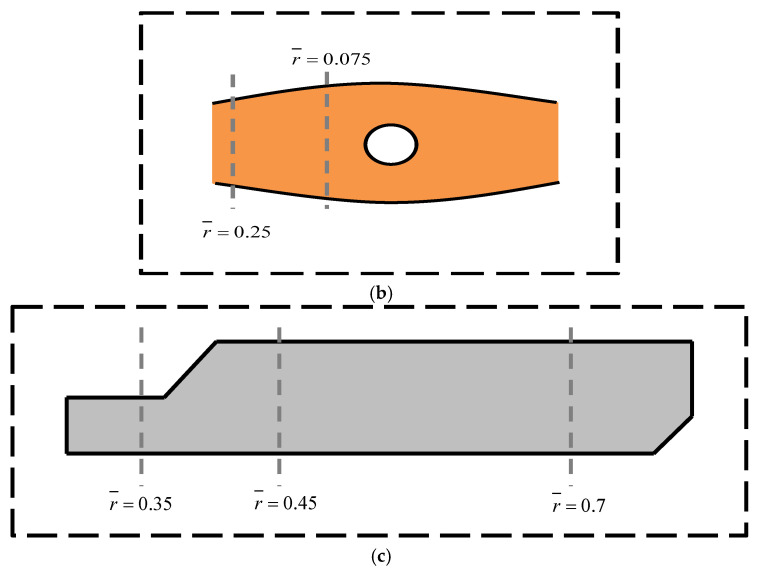
(**a**) The test points of the tail rotor pitch link. (**b**) The test points of blades. (**c**) The test points of the flexbeam.

The test points of the tail rotor pitch link, the tail rotor blades and the flexbeam are shown in Figure 4. And the load types at critical test points are shown in Table 2. According to Table 2 and Figure 4, the strain orthogonal gauges were mounted on the tail rotor pitch link to measure axial load; the strain gauges were mounted on the flexbeam $\bar{r}=\frac{r}{R}=0.075,0.25$ or the tail rotor blades $\bar{r}=0.35,0.45,$ 0.7 to measure bending moments, among which, according to [1], the lagwise bending moments were measured using a decoupling method.

### 3.2. Testing Equipment

The helicopter tail rotor system is one of the most important components of a helicopter, which exhibits characteristics of high-speed rotation and complex dynamic behavior, so the TR (tail rotor) Signal Collector should exhibit the following properties. Firstly, TR Signal Collector can operate normally in environments with high-speed rotation and severe vibration conditions. Secondly, the system shall be equipped with multi-channel data acquisition, recording and telemetry capabilities, while maintaining synchronization with the helicopter’s BeiDou satellite time signals. Based on engineering experience, typical Data Acquisition Systems should have a minimum sampling rate of 1024 Hz and time synchronization accuracy better than 1 ms. Finally, to ensure flight, the TR Signal Collector must be lightweight and minimally disruptive to the helicopter’s aerodynamic characteristics. In this study, the weight of TR Signal Collector is designed in accordance with MIL-STD-881F, and the helicopter modification is performed in strict compliance with MIL-STD-881F and ADS-51-HDBK.

**Figure 5:**
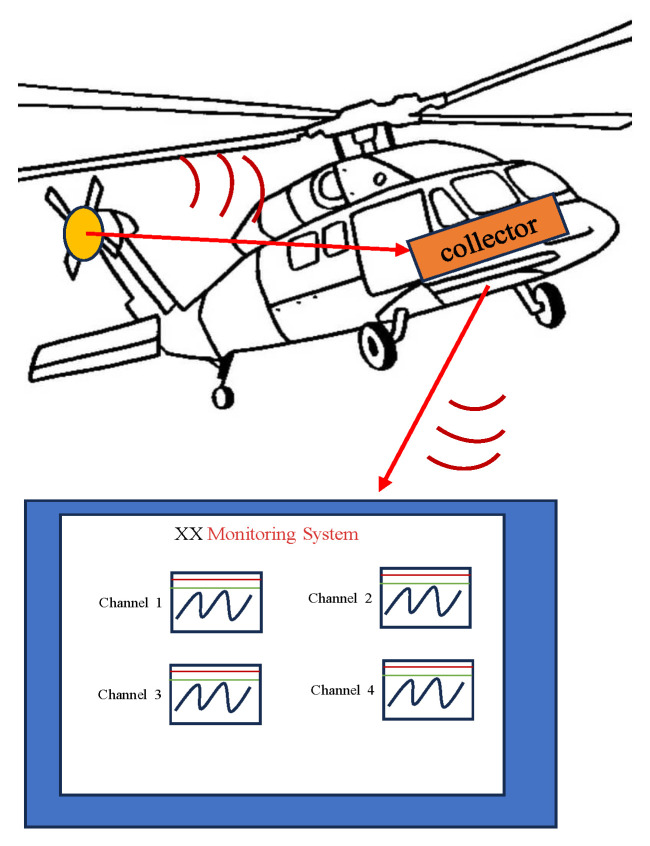
TR acquisition system.

TR acquisition system consist of three main components, as shown in Figure 5. The first part is the TR-mounted probe; the second part is the airborne testing system mounted in the helicopter cabin; the third part is the real-time bi-directional telemetry of the mission operations room. During standard helicopter operations, aerodynamic and inertial loads acting on the tail rotor induce structural deformation. These strain variations are measured by bonded resistance strain gauges, with the resultant electrical signals being conditioned and logged by the tail rotor Data Acquisition System (DAS). The DAS is composed of modules such as data recording, storage, timing and telemetry. The sampling rate of the collector is set to 1024 Hz.

**Figure 6:**
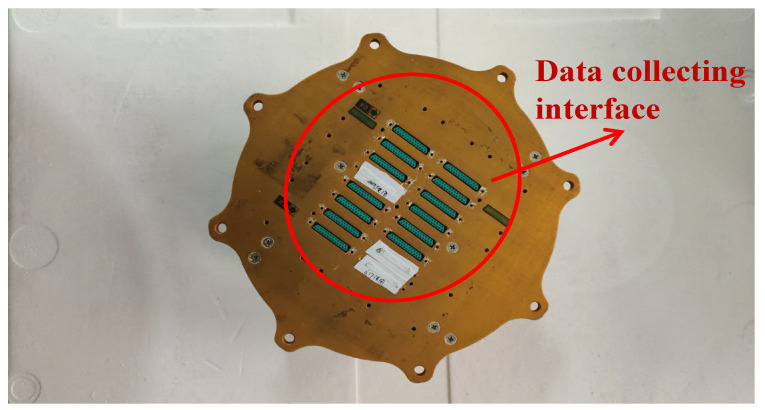
TR Signal Collector.

TR Signal Collector is the most important part in the TR Acquisition System, which serves as the source for load data acquisition and recording. TR Signal Collector is shown in Figure 6. The green area in Figure 6 indicates the data acquisition channel interfaces.

The TR Signal Collector for flight testing records load data using encoded values. The encoded value range is 0~65535. The excitation voltage of the TR Signal Collector is *E* = ±5 V and the output voltage of that is *U* = ±20 mv. The Wheatstone bridge formula is the following:

(1) $$△U=\frac{EK\varepsilon }{4}$$

**Figure 7:**
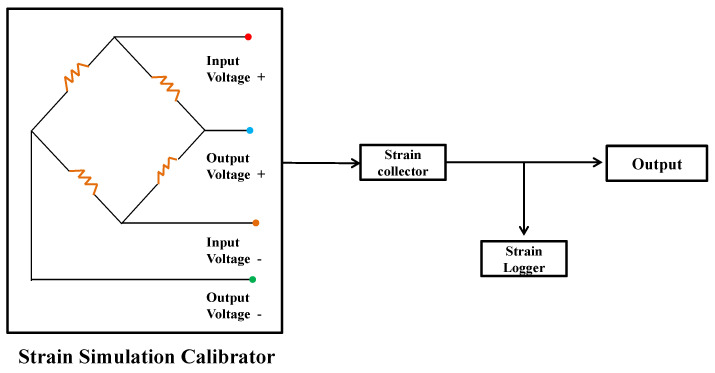
The standard strain simulator system.

**Figure 8:**
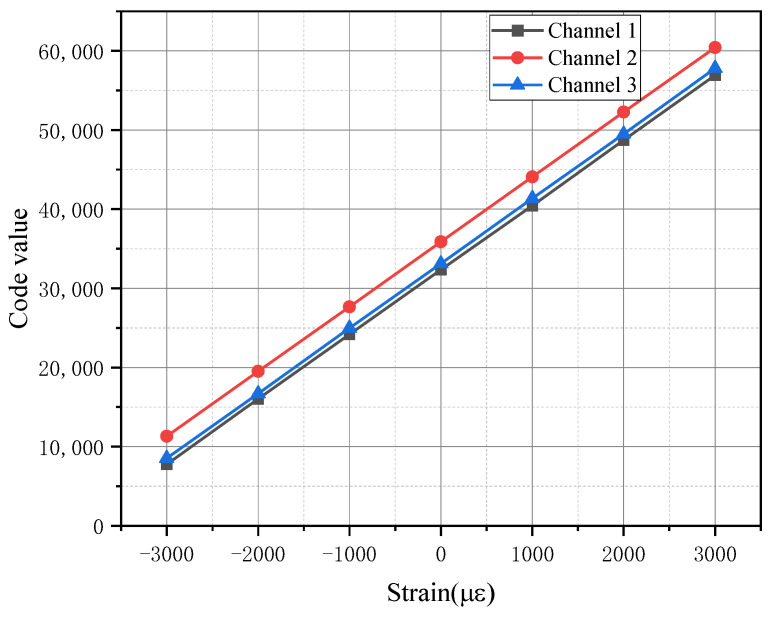
Channel curves of TR (tail rotor) Signal Collector.

△U  is the varying voltage, *K* is the gauge factor of strain gauge, typically taken as *K* = 2.11. According to Formula 1, the theoretical range of the collector is ±4000 με. The correlation coefficient between the code value and strain is $K^{\prime }=0.1221\;\mu \varepsilon$. The channel was calibrated using a standard strain simulator, and the calibration schematic is shown in Figure 7. During strain calibration, the test system bridge circuit must be disconnected and replaced with a strain simulator. The resistance of a single arm of the calibrator is then adjusted to simulate the strain bridge variations experienced on the helicopter. The calibration results of three selected channels are presented in Figure 8. As illustrated, the three channels exhibit nearly identical slopes while showing different intercepts. This discrepancy arises from inherent zero-offset differences between individual channels in the Acquisition System. Theoretically, the system’s zero-reference point corresponds to a digital output code of 32767.

### 3.3. The Measurement Errors

The measurement errors in this work are dominated by the following four aspects:

First, error from the ground load calibration test. In Section 3, strain gauges configured in a Wheatstone bridge were adopted to conduct load calibration, and the strain–load relationship was established accordingly. The test design and results demonstrate that the load calibration curves exhibit excellent repeatability, with a linear fitting accuracy of up to 99%, indicating a negligible error from the ground calibration test. The residual errors mainly originate from the instrumental error of the KM500 acquisition unit and the inherent error of strain sensors, both of which fall within the allowable range for engineering applications. According to long-term engineering experience in load flight tests, the overall combined error is approximately 3%.

Second, error induced by the helicopter vibration environment. Such an error can only be mitigated through structural optimization and proper installation of the Acquisition System, and cannot be quantified precisely. The detailed measures to suppress this error are addressed in the response to the sixth comment.

Third, error evaluation based on post-flight data analysis. The measured data present favorable periodicity with few abnormal spikes, and the Fourier transform results are consistent with the rotation period of the tail rotor blades, verifying the reliability of the measured data.

Finally, comparison with design data. The measured loads have been compared with the design values provided by the design institution, and satisfactory agreement has been obtained and recognized by the design institution. The proprietary design data are not available for public disclosure here.

## 4. Ground Load Calibration Test

Prior to conducting flight tests, ground load calibration tests must be performed to establish the relationship between applied loads and strain responses. Based on the strain data measured during actual flight tests, the operational loads acting on the helicopter tail rotor components can then be inversely calculated. Therefore, the accuracy of ground load calibration tests directly determines the reliability of operational load measurements obtained during flight test.

### 4.1. Ground Load Calibration Test for the Tail Rotor Pitch Link

**Figure 9:**
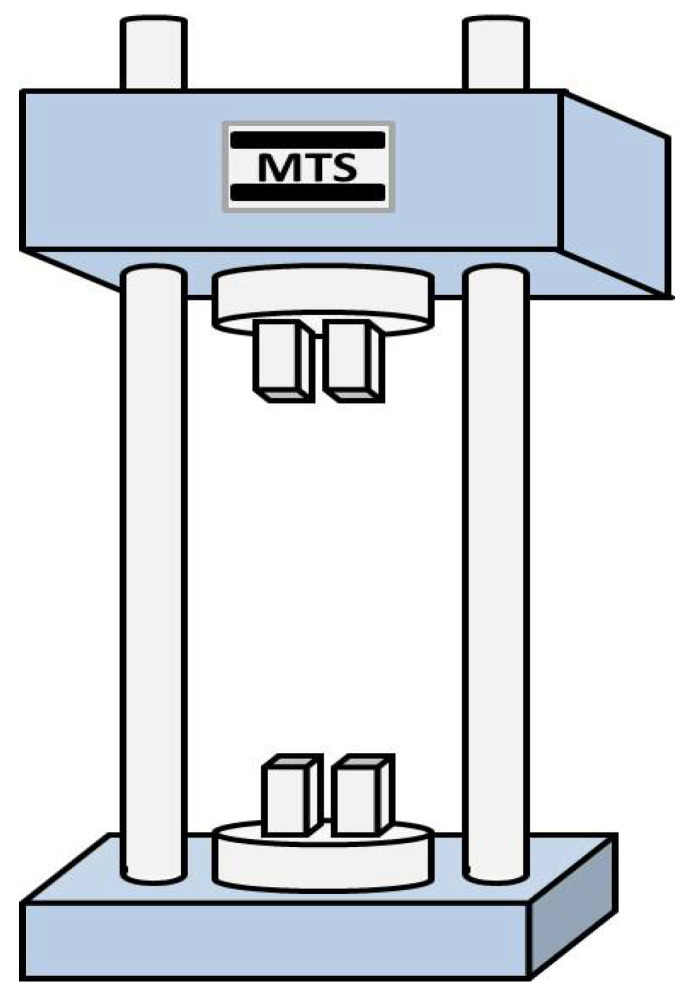
The MTS universal testing machine.

Tail rotor pitch link is the two-force member, whose load calibration test is relatively straightforward. Firstly, according to the load limits *F_N_* specified in the design, the maximum load applied during ground testing was 80% of *F_N_*. In this test, the tail rotor pitch link was subjected to tension and compression testing using a five-stage load loading protocol, 0, 20%, 40%, 60%, 80%, 100% maximum load, the maximum, with three complete test cycles performed. Secondly, the calibration coefficient *k* for the relationship is obtained fitting the experimental data points from the *it*h test. Finally, the final calibration coefficient $\bar{k}=\sum_{i=1}^{6}k_{i}$ is obtained by averaging the series of individually determined coefficients from multiple test cycles. This calibration test was conducted using an MTS universal testing machine, with detailed technical specifications provided in Table 3 and a schematic diagram of the loading setup shown in Figure 9.

**Figure 10:**
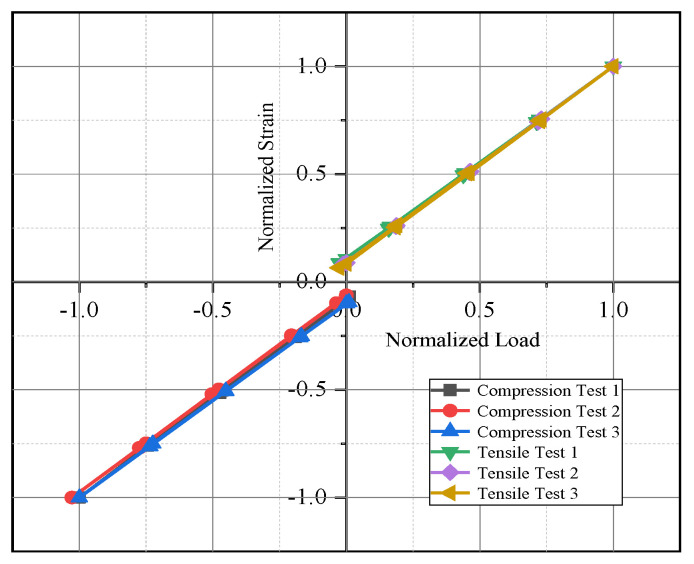
The results of load calibration on the tail rotor pitch link.

Load calibration on the tail rotor pitch link is based on the designed test, and the results are shown in Figure 10. As shown in Figure 10, the load–strain relationship coefficients obtained from both tensile and compressive calibrations are essentially consistent, indicating that the experimentally measured data are reliable.

### 4.2. Ground Load Calibration Test for the Tail Rotor Blades

**Figure 11:**
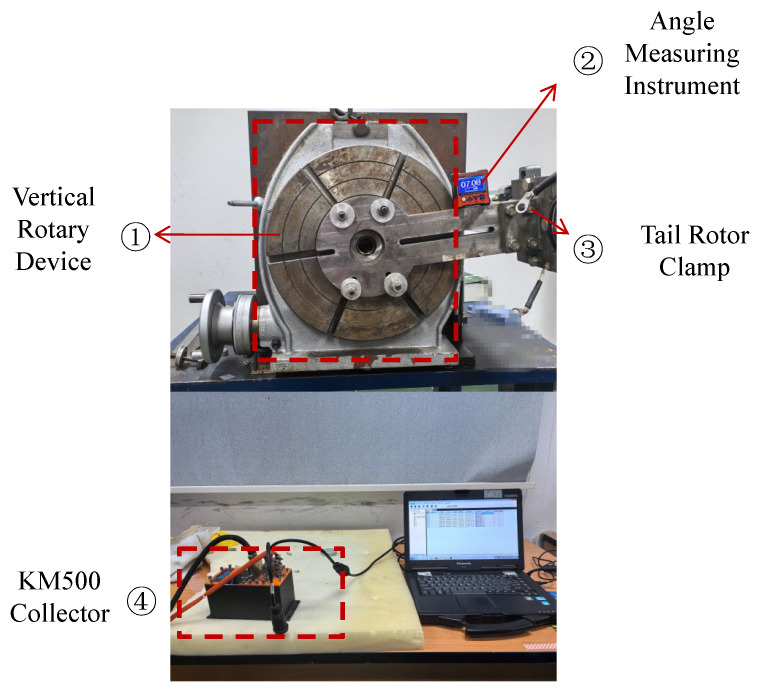
The vertical rotating device and KM500 collector.

According to the structure of the tail rotor blade, it can be equivalent to a cantilever beam. During normal helicopter operation, any cross-section of the tail rotor blade is subjected to bending moments, torque generated by aerodynamic forces and centrifugal loads caused by rotation. For the helicopter flight testing, the primary focus is on fatigue loads of dynamic components. Since the rotor speed remains essentially constant during steady helicopter operation, the centrifugal force ($F_{L}=m\omega^{2}r$) can be considered invariable. Therefore, centrifugal force does not need to be measured directly in flight tests. The same occurs for the calibration test of the tail rotor pitch link, firstly, according to the load limit specified in the design, flapwise bending moment *M_H_*, lead-lag bending moment *M_B_* and torque *T_Q_*. The maximum load applied during ground testing is 80%. In this test, the tail rotor blades were subjected to load using a five-stage load loading protocol. The vertical rotating device was used to complete this test. Loading and data acquisition equipment is shown in Figure 11.

The 1 is the vertical rotary device; the 2 is the angle measuring instrument; the 3 is the tail rotor clamp; the 4 is the KM500 collector.

**Figure 12:**
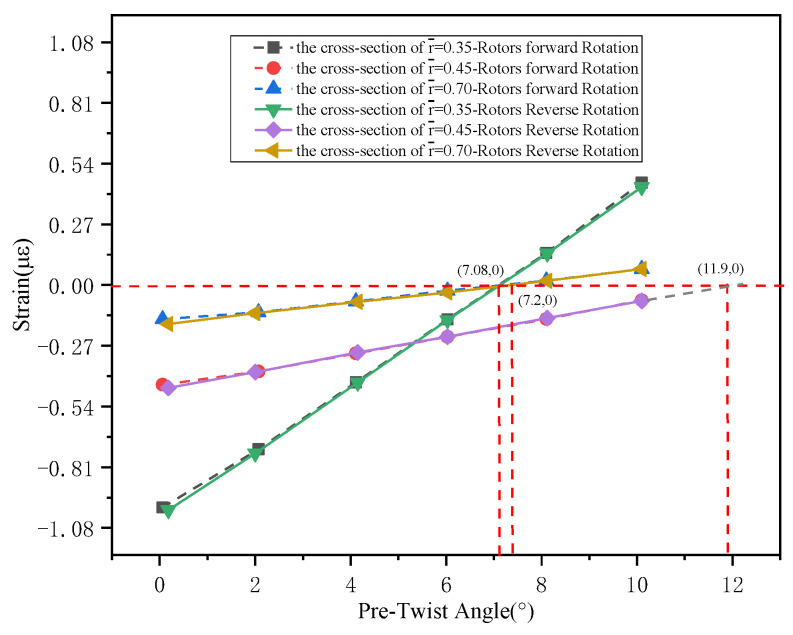
The results of pre-twist angle.

Since the tail rotor blade is not a standard symmetric cantilever beam, it is necessary measure the “equivalent neutral plane” for each cross-section before conducting load calibration tests (to ensure that lead-lag loading does not induce flapwise strain). The measurement method follows ref. [22], and the test results are shown in Figure 12. The pre-twist angle of section $\bar{r}=0.35$ is 7.08°; the pre-twist angle of section $\bar{r}=0.45$ is 7.2°; the pre-twist angle of section $\bar{r}=0.7$ is 11.9°.

**Figure 13:**
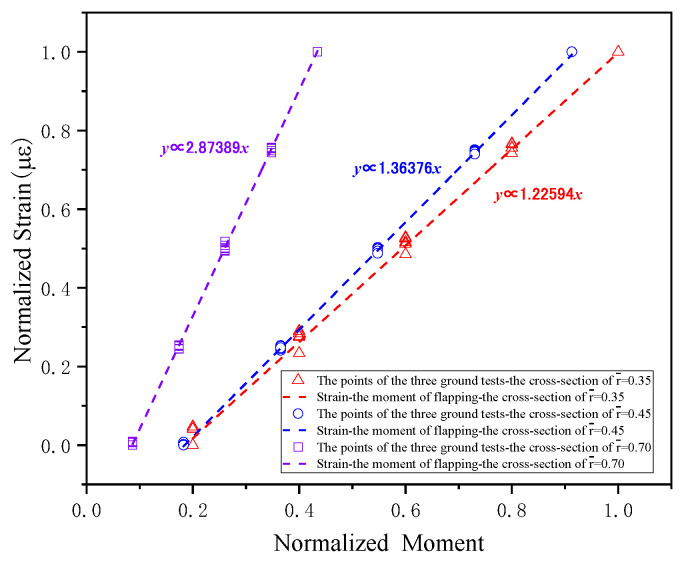
The results of flapwise bending moment on the blade cross-sections.

**Figure 14:**
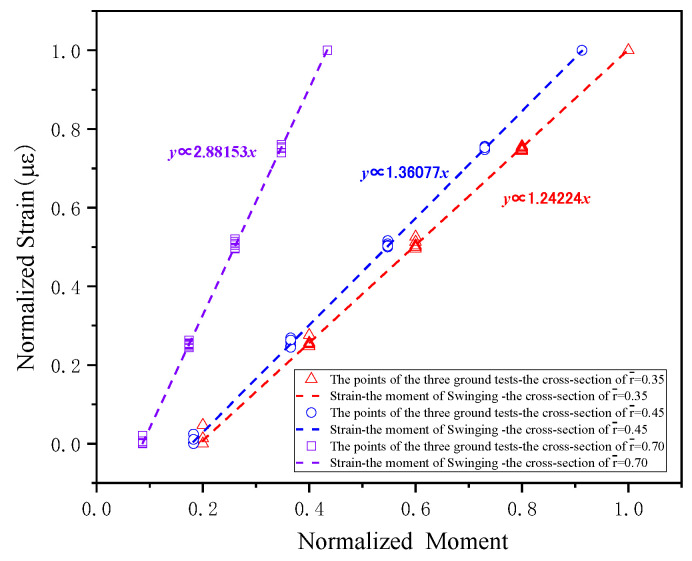
The results of lead-lagwise bending moment on the blade cross-sections.

**Figure 15:**
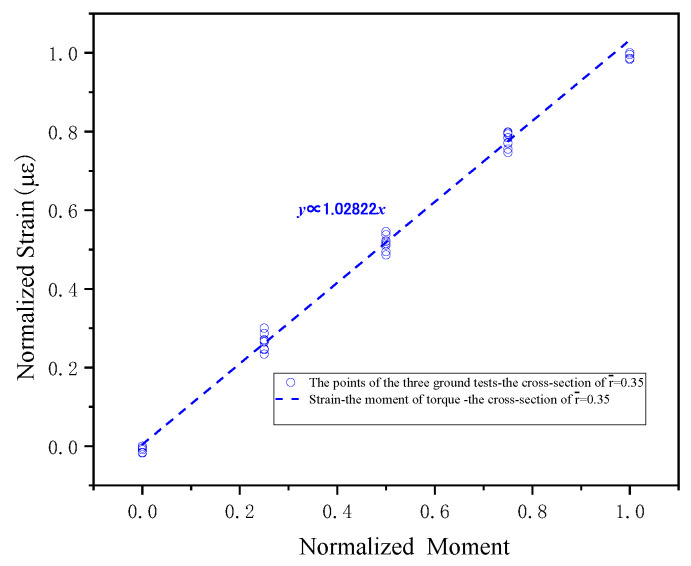
The results of torque moment on the blade cross-section.

The load calibration has been performed for each blade cross-section based on the aforementioned test results, with calibration outcomes presented in Figure 13, Figure 14 and Figure 15.

The load-to-digital conversion coefficient (K) can be obtained by combining the strain-to-digital coefficient ($K^{\prime }$) from ground calibration tests with the load-to-strain coefficient ($\overset{-}{k}$) from ground load calibration tests. During subsequent flight testing, the load values acting on the tail rotor system in flight can be determined based on the measured digital values.

## 5. Flight Test Designing and Loading Analysis

Prior to conducting flight tests, the flight test maneuvers must be carefully designed. The flight test maneuvers that induce severe loads on tail rotor components shall be selected for testing, as detailed in Table 4.

Flight test measurement parameters is shown in Table 5.

Flight tests were conducted based on the designed maneuver syllabus, and the tail rotor component loads were measured under various flight test conditions.

### 5.1. Sideward/Backward Flight

**Figure 16:**
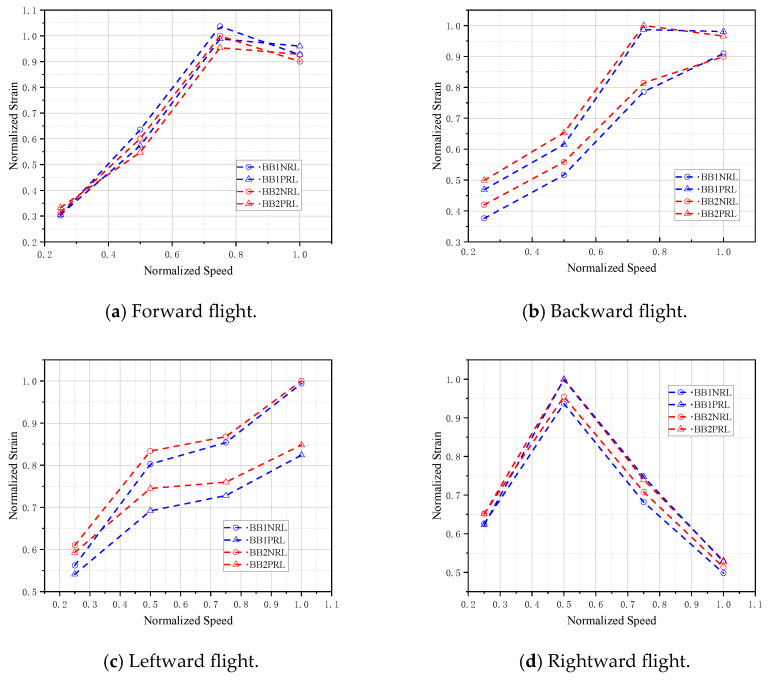
Bending moment of the tail rotor shaft during helicopter sideward/backward flight.

The tail rotor load diagram of a helicopter in a sideward/backward flight condition is shown in Figure 16. As can be seen from the figure, during forward flight, backward flight, and leftward flight, the bending moment load generally exhibits a gradually increasing trend, whereas during rightward flight, the bending moment load tends to gradually decrease.

When the helicopter performs forward flight or backward flight, as the speed increases, the power required gradually rises, leading to an increase in the power allocated to the tail rotor, which in turn causes higher tail rotor loads. However, during forward flight at $\bar{V}=0.8$, a load reduction occurs because this speed corresponds to the helicopter’s transitional flight regime, where the tail rotor operates in an unfavorable aerodynamic environment, resulting in higher loads.

For a helicopter with the tail rotor mounted on the right side of the fuselage, during leftward flight, an increase in speed requires higher tail rotor thrust, leading to an increase in tail rotor load. Conversely, during rightward flight, an increase in speed requires reduced tail rotor thrust, resulting in a decrease in tail rotor load. At $\bar{V}=0.5$, a load inflection point is observed, which may be related to the vortex ring state of the helicopter’s tail rotor.

### 5.2. Level Flight

**Figure 17:**
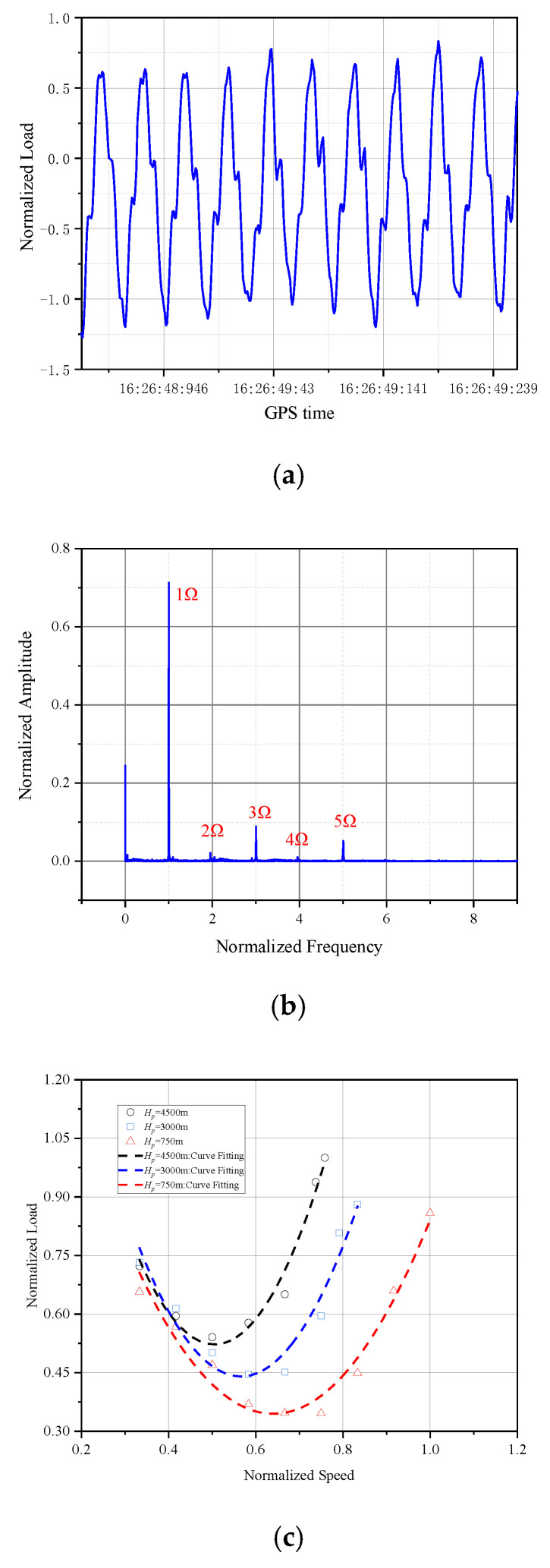
(**a**) Real-time load curve. (**b**) The FFT of the load in tail rotor. (**c**) Tail rotor load in level flight at different altitudes.

As shown in Figure 17a,b, during level flight, the tail rotor load curve exhibits good periodicity with no noise interference. The tail rotor load is analysis by FFT method, which shows that dominant load frequencies are integer multiples of the tail rotor speed, confirming the authenticity and validity of the recorded load data.

The tail rotor loads in level flight at different altitudes is shown in Figure 17c. The load–speed curve exhibits a saddle-shaped profile, where the tail rotor load initially decreases and then increases with speed. This trend indicates a correlation between load variation and engine power output. At the same speed, the tail rotor load increases with rising pressure altitude. This phenomenon occurs because reduced air density at higher altitudes necessitates greater tail rotor pitch angles to maintain torque equilibrium, consequently increasing tail rotor loads.

### 5.3. Sawtooth Climb

**Figure 18:**
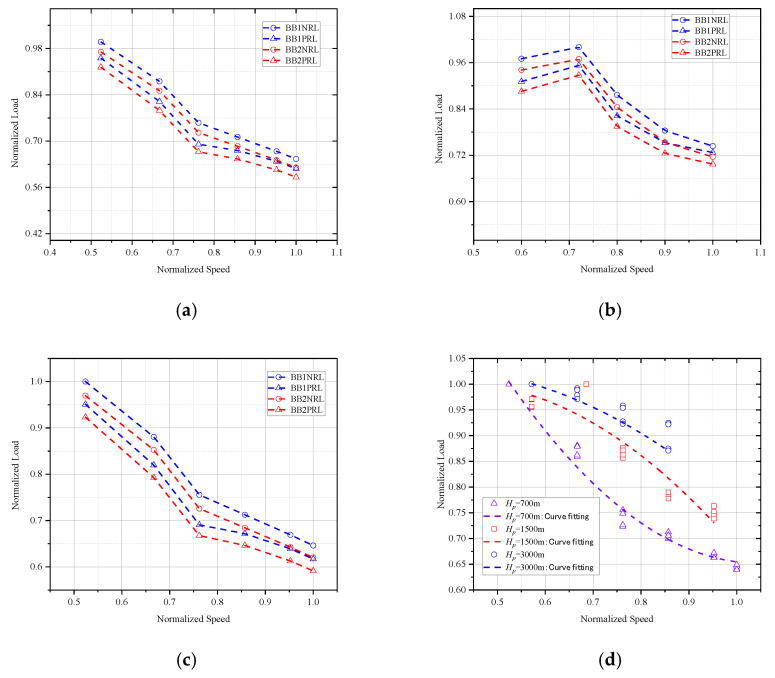
(**a**) *H_p_* = 700 m: The tail rotor load of sawtooth climb maneuver (**b**) *H_p_* = 1500 m: The tail rotor load of sawtooth climb maneuver. (**c**) *H_p_* = 3000 m: The tail rotor load of sawtooth climb maneuver. (**d**) Speed–load curve at different altitudes.

The tail rotor load is shown in Figure 18 during sawtooth climb maneuvers. As illustrated in Figure 18a–c, the tail rotor load progressively diminishes with increasing airspeed. This phenomenon occurs because during the sawtooth climb maneuver, while the helicopter’s engine maintains constant power output, the augmented velocity reduces the available power distribution to the tail rotor system, consequently decreasing tail rotor loading.

As evidenced in Figure 18d, the tail rotor load increases with rising pressure altitude due to decreasing air density. To keep helicopter attitude stability in these conditions, greater power must be allocated to the tail rotor system, resulting in elevated tail rotor loading.

### 5.4. Near-Ground Maneuvers

#### 5.4.1. Obstacle Slalom Maneuver

Obstacle Slalom Maneuver is one of the standard test maneuvers specified in ADS-33E-PRF [23], primarily designed to evaluate:

First, a helicopter’s agile maneuvering capability relative to ground obstacles;

Second, turn coordination during dynamic flight;

Third, cross-axis coupling intensity between control channels.

**Figure 19:**
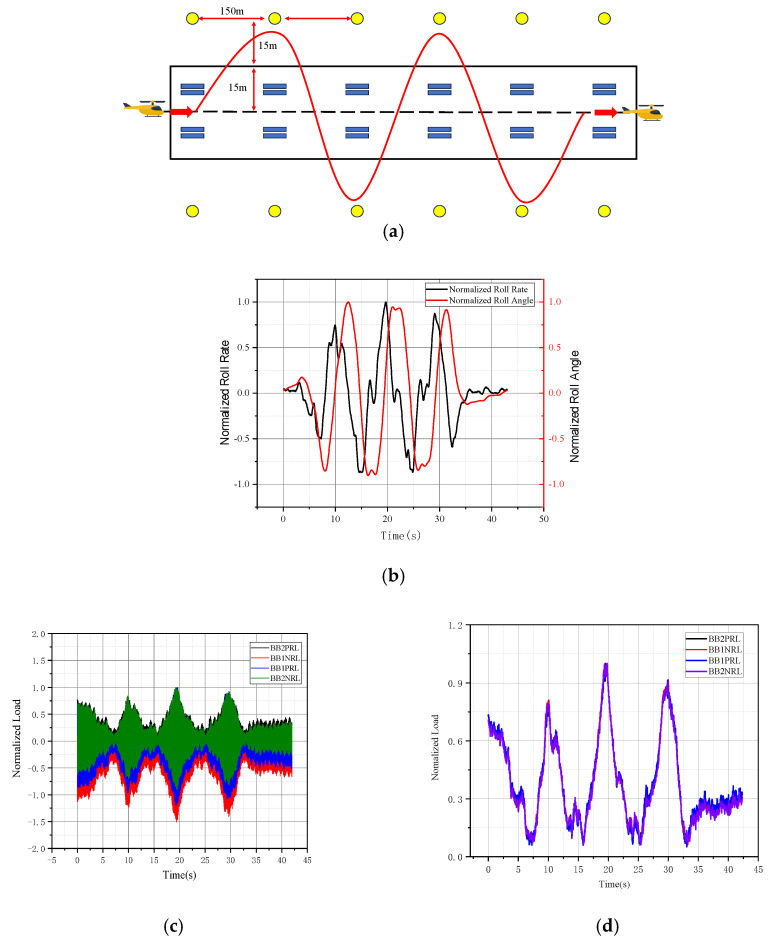
(**a**) Obstacle Slalom Maneuver schematic diagram. (**b**) The helicopter’s rolling ability. (**c**) The real-time load of Obstacle Slalom Maneuver. (**d**) The real-time dynamic load of Obstacle Slalom Maneuver.

When the helicopter (with the tail rotor mounted on the right side) enters the Obstacle Slalom Maneuver phase, its indicated IAS remains essentially constant. During right or left turns, the pilot must increase or reduce collective pitch to maintain maneuver performance. The roll angle and roll rate exhibit periodic variations (shown in Figure 19b), indicating that the pilot’s control inputs comply with standard procedures and the measured load data is reliable and representative.

The real-time load and dynamic load of Obstacle Slalom Maneuver, which also exhibit periodic variation characteristics, are shown in Figure 19c,d. Dynamic load increases with rising roll rate and decreases with reducing roll rate. Dynamic load peaks occur at approximately 10 s, 20 s, and 30 s, while troughs appear at 7 s, 15 s, and 25 s. Higher roll rate peaks correlate with increased dynamic load peaks. When the helicopter (with the tail rotor on the right side of the fuselage) enters obstacle, the main rotor power distribution increases, and the load increases when making a right turn. Conversely, when making a left turn, the tail rotor load decreases.

#### 5.4.2. Deceleration to Sprint Maneuver

**Figure 20:**
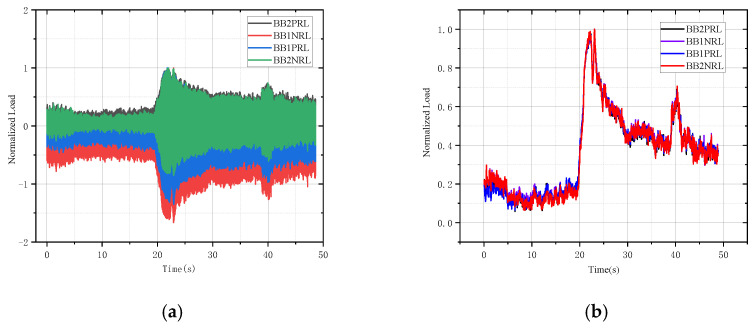
(**a**) The real-time load of deceleration to sprint maneuver. (**b**) The real-time dynamic load of deceleration to sprint maneuver.

When the helicopter is performing deceleration to sprint maneuvers, the dynamic load is shown in Figure 20. The deceleration stage of the helicopter is 0~20 s, during which the engine power output is low, resulting in a low tail rotor load. During the helicopter sprint phase from 20 s to 25 s, the torque of the helicopter engine increases sharply, and the gauge speed also increases in a short period of time. The tail rotor also shows a sharp increase in response. After 25 s, the helicopter changed to this action, and the torque of the helicopter gradually stabilized, while the tail rotor load showed a decreasing trend.

Based on the classic maneuver load analysis, a helicopter load risk test point matrix can be derived, as shown in Table 6. Flight test profiles (e.g., left flight, right flight, deceleration to sprint) is in the first column. Helicopter attitudes (e.g., low IAS, high IAS, low altitude) is in the first row. Cells marked with yellow background and red stars (★) denote high-risk load test points under specific combinations of flight profiles and attitudes (e.g., the cell at Row 2, Column 3 indicates that high-speed left flight is a critical risk point). This test point matrix can provide data support flight test monitoring, reduce the risk of flight tests, and effectively increase monitoring efficiency, reducing the manpower, material resources, and financial resources required for monitoring.

## 6. Conclusions

After analyzing the tail rotor load of classic helicopter flight maneuvers, the following three conclusions can be drawn:

Firstly, the parameters and patterns that affect the tail rotor load vary among different flight maneuvers of helicopters. During leftward flight, the tail rotor load increases with IAS, whereas during rightward flight, the tail rotor load decreases as airspeed rises.

Secondly, developing a helicopter load risk test point matrix can provide a critical reference for flight test monitoring, effectively reducing flight test risks while enhancing monitoring efficiency and cutting down on manpower, material, and financial costs.

Thirdly, the helicopter maneuvers discussed in this study are relatively limited. To support comprehensive engineering testing, it is necessary expand the flight test envelope by incorporating additional test profiles, thereby establishing a complete risk point matrix to effectively guide flight test operations.

## Figures and Tables

**Table 1:** The fundamental parameters of the strain gauges.

Manufacturer	Model	Substrate	Resistance Value (Ω)	Gauge Factor
AVIC ZEMIC	BA350-2AA(9)-G150-JQC	Metal Foil	350 ± 0.2	2.11 ± 0.01

**Table 2:** The load types of the test points.

	Test Points	Axial Load	Flapwise Bending Moment	Lagwise Bending Moment	Torque
Load Types					
The center of the tail rotor pitch link		√			
Flexbeam $\bar{r}=0.075$			√	√	
Flexbeam $\bar{r}=0.25$			√	√	
The tail rotor blades $\bar{r}=0.35$			√	√	√
The tail rotor blades $\bar{r}=0.45$			√	√	
The tail rotor blades $\bar{r}=0.7$			√	√	

**Table 3:** The key parameters table of the MST universal testing machine.

Max Force	Accuracy Specifications	Resolution Specifications	Note
10 kN	±1% FS	0.02%	-

**Table 4:** The flight test points.

Serial Number	Pressure Altitude	Maneuver
1	0~1000 m	Level Flight
2	2000 m~3000 m	Level Flight
3	3000 m≥	Level Flight
4	Near-Ground	Sideward/Backward Flight
5	700 m	Sawtooth Climb
6	1500 m	Sawtooth Climb
7	3000 m	Sawtooth Climb
8	Near-Ground	Deceleration to Sprint
9	Near-Ground	Slalom

**Table 5:** Flight test measurement parameters.

Serial Number	Parameter Type	Symbol	Sampling Rate (Hz)
1	Pressure/Radio Altitude	m	32
2	Static Air Temperature (SAT)	℃	32
3	Indicated Airspeed (IAS)	km/h	32
4	Collective Position	mm	32
5	Roll Angle Rate	(°/s)	32
6	Pitch Angle Rate	(°/s)	32
7	Yaw Angle Rate	(°/s)	32
8	Roll Angle	(°)	32
9	Pitch Angle	(°)	32
10	Yaw Angle	(°)	32
11	Engine Torque	%	32

**Table 6:** The helicopter load risk test point matrix.

	Low IAS	High IAS	Low Altitude	High Altitude	Large Attitude	Small Attitude	……
Leftward Flight		★					
Rightward Flight	★						
Backward Flight		★					
Level Flight		★		★			
Sawtooth Climb	★			★			
Near-Ground Maneuver					★		
⋮							
red stars (★) denote high-risk load test points under specific combinations of flight profiles and attitudes							

## Data Availability

The data in this article is not provided due to privacy concerns.

## List of Abbreviations

Fiber Bragg Grating	**FBG**
Tail Rotor	**TR**
Data Acquisition System	**DAS**

## References

[1] Leishman J.G. (2006). Principles of Helicopter Aerodynamics.

[2] Johnson W. (2022). Advanced rotorcraft load measurement techniques. AIAA J..

[3] Kim H., Lee S. (2023). High-speed FBG sensing for tail rotor loads. Meas. Sci. Technol..

[4] Zhang M., Li H., Wang J. (2023). Dynamic load measurement of helicopter tail rotor using FBG. Chin. J. Aeronaut..

[5] Smith R., Brown T. Machine Learning for Tail Rotor Load Prediction. Proceedings of the AIAA Aviation Forum.

[6] FAA (2023). Advisory Circular 29-2D: Transport Category Rotorcraft Certification.

[7] NASA (2023). CR-202301256: Wireless Rotor Load Monitoring.

[8] Airbus Helicopters (2023). H160 Tail Rotor Flight Test Report (Internal Rep. AH-TR-2305).

[9] Schmidt T. (2020). Uncertainty analysis in strain gauge measurements under dynamic loading. Meas. Sci. Technil..

[10] Zhang H.L., Wang Z.F., Teng F., Xia P.Q. (2023). Dynamic strain measurement of rotor blades in helicopter flight using fiber bragg grating sensor. Sensors.

[11] Deng F.W. (2025). Analysis on flight test load characteristics of main rotor for large helicopter. China Sci. Technol. Inf..

[12] Zheng J.H., Ma D.F., Jiao S.K. (2021). Flight test research on load measurement of helicopter rotor shaft. China Meas. Test.

[13] Wang Z.F., Song R.X., Zheng J.H. (2026). A Rotor Aerodynamic Load Analysis Method Based on Measured Structural Load.

[14] Wu H.F., Dong R. (2022). Research on helicopter rotor blade load model based on neural network. J. Guilin Univ. Aerosp. Technol..

[15] Zhang J.N., Yu J.J., Chen Y.P. (2026). A Helicopter Rotor Flight Load Prediction Method Based on RBF Neural Network.

[16] Liu M.M., Liu B.K., Dai Q. (2020). Load measurement methods for helicopter rotor blades. Electron. Technol. Softw. Eng..

[17] Cheng W.Z. (2019). Flight test research on structural load of coaxial dual rotor blades. Chin. J. Appl. Mech..

[18] Zhang H.L., Cheng W.Z., Xia P.Q. (2025). Engineering modeling and flight test verification of rotor dynamic load based on distributed FBG sensing measurement. Chin. J. Appl. Mech..

[19] Zhu H., Hu S., Zhang L., Li M., Zhu R. (2024). Dynamic Characteristics of a Supercritical Helicopter Tail Transmission System with Self-Excited Vibration and Rubbing Impact. Int. J. Struct. Stab. Dyn..

[20] Brown K. (2022). Strain gauge arrays for load monitoring in wind turbine blades. Meas. Sci. Technil..

[21] Smith J. (2021). High-frequency strain measurement using piezoresistive strain gauges. Meas. Sci. Technil..

[22] Jiao S., Geng L., Wang Z. (2022). Load calibration test technology of rotor under blade gravity. China Meas. Test.

[23] U.S. Army Aviation and Missile Command (2021). Aeronautical Design Standard-33E-PRF: Handling Qualities Requirements for Military Rotorcraft Redstone Arsenal.

